# Potential Health Benefits of Deep Sea Water: A Review

**DOI:** 10.1155/2016/6520475

**Published:** 2016-12-26

**Authors:** Samihah Zura Mohd Nani, F. A. A. Majid, A. B. Jaafar, A. Mahdzir, M. N. Musa

**Affiliations:** ^1^Malaysian-Japan International Institute of Technology, Universiti Teknologi Malaysia, 54100 Kuala Lumpur, Malaysia; ^2^UTM Ocean Thermal Energy Centre (OTEC), Universiti Teknologi Malaysia, 54100 Kuala Lumpur, Malaysia; ^3^Tissue Culture Engineering Research Laboratory, Faculty of Chemical Engineering, Universiti Teknologi Malaysia, 81310 Skudai, Johor, Malaysia; ^4^Institute of Marine Biotechnology, Universiti Malaysia Terengganu, 23000 Kuala Terengganu, Terengganu, Malaysia; ^5^Perdana School of Science, Technology, Innovation and Policy, Universiti Teknologi Malaysia, 54100 Kuala Lumpur, Malaysia

## Abstract

Deep sea water (DSW) commonly refers to a body of seawater that is pumped up from a depth of over 200 m. It is usually associated with the following characteristics: low temperature, high purity, and being rich with nutrients, namely, beneficial elements, which include magnesium, calcium, potassium, chromium, selenium, zinc, and vanadium. Less photosynthesis of plant planktons, consumption of nutrients, and organic decomposition have caused lots of nutrients to remain there. Due to this, DSW has potential to become a good source for health. Research has proven that DSW can help overcome health problems especially related to lifestyle-associated diseases such as cardiovascular disease, diabetes, obesity, cancer, and skin problems. This paper reviews the potential health benefits of DSW by referring to the findings from previous researches.

## 1. Introduction 

Water is generally defined as a liquid which is shaped by the container that it is filled in and is able to have many variants of colours. It is the crucial component for all living things. For instance, humans need water for many functions such as to regulate body temperature, enhance body metabolism, and provide minerals that are essential to the body. There are many sources of water, such as surface water, aquifer, spring, and seawater. Meanwhile, deep sea water (DSW) can also be a good water source. It is beneficial as it could supply minerals that are essential to health. DSW commonly refers to seawater that is pumped up from a depth of over 200 m. It is usually associated with the following characteristics: low temperature, high purity, and being rich in nutrients, namely, beneficial elements [1]. Its location being far from solar radiation results in it having minimal to no bacteria activities. Less photosynthesis of plant plankton, consumption of nutrients, and lots of organic decomposition causes abundant nutrients to remain there. The abundance of inorganic material becomes higher when the depth of the seawater is increased. These characteristics have derived attention for research regarding DSW especially for its numerous beneficial minerals, which include magnesium (Mg), calcium (Ca), potassium (K), chromium (Cr), selenium (Se), zinc (Zn), and vanadium (V) [1, 2]. DSW is claimed to be high in minerals compared to other sources of water [2].

People usually consume drinking water that is in the form of bottled drinking water (such as mineral water), filtered tap water, or boiled tap water. Drinking water sold by suppliers is expected to contain good nutrient content and be safe to be consumed, because the suppliers possess a production license from the authorities. Surprisingly, some drinking water that is available in the market has been reported to have low mineral content [3]. This is possibly due to the common process drinking water undergo such as reverse osmosis and filtration, which removes the mineral contents inside it. Mineral water, which does not undergo the extensive process needed, is completely taken from groundwater and gains mineral ions from its sources such as rocks. It is also reported to contain low minerals [3]. However, the mineral contents in the water may vary with the geographical locations and the treatment process that it has gone through. Promisingly, DSW can offer plenty of minerals for the production of drinking water, and other DSW by-products. The production of refined DSW usually involves a desalination process, followed by a mineralization process. A high concentration of mineral salts in DSW though will commonly be processed through means such as reverse osmosis, electrodialysis, or low vacuum temperature in order to produce a safe concentration of water for consumption [1, 4, 5].

DSW has been acquired from many countries with sources of it that are accessible to land. This include Korea, Japan, Taiwan, China, and the USA [1, 2, 6, 7]. Most of those countries conducted researches regarding the health effects that can be attained from the consumption of DSW. As a result, the production of products such as deep sea drinking water (DSDW) became available from those countries. DSDW is claimed as a drinking water which can promote health, since it does not contain carbohydrate, fat, protein, and other bioactive materials which potentially cause adverse health effects, instead of providing valuable minerals to health. Despite being produced for drinking water, it is also used for a variety of purposes such as for food products, cosmetics, aquaculture, and agriculture [8]. Thus, due to the availability of numerous minerals, many researches have been conducted regarding it, in order to discover its benefits to health. By conducting literature reviews, the findings regarding the potential health benefits of DSW applications have been compiled and discussed in this paper.

## 2. Minerals in Deep Sea Water

DSW contains many types of minerals, such as Mg, Ca, Cl, Na, K, Se, and V, as shown in Table 1 [8]. In fact, DSW is more abundant in minerals compared to surface seawater [6]. The example of the difference between the amount of minerals among surface seawater and DSW is shown in Table 2. DSW is a good nutrient source and could be claimed as a nutrients provider, since the minerals contained inside it provide many benefits to health. For instance, Mg is significant for many physiological processes in the body such as for energy metabolism and enzyme functions [9]. Mg is able to reduce lipids accumulation in the aorta of subjects that has high cholesterol intake [10]. Besides that, Mg is beneficial to people who have cardiovascular disease as it can reduce the potential of a heart attack by dilating the blood vessels and stopping spasms in the heart muscles and vessel walls [11]. It is also able to reduce the risk of obesity, diabetes, and asthma [1, 12]. Drinking water, which has high Mg content, has shown higher inhibitory effects in the adipocyte differentiation, which means that the synthesis of fat cells is able to be slowed down by Mg [13]. Ca is one of the major minerals for humans. It has many benefits to health such as for bone development and density and acts as the pivotal cofactor for several enzymes needed for energy metabolism. Adequate intake of Ca can help reduce the risks of cardiovascular disease, obesity, and some forms of cancers [1, 9, 12]. A high Ca diet is able to increase lipolysis and preserve thermogenesis during caloric restriction, in a way that markedly accelerates weight loss [14]. Cr is an essential nutrient that is required for carbohydrates and lipids metabolism [15, 16]. It has antioxidant properties which are useful for expanding cell life [17]. V has the potential for reducing lipids and has shown effectiveness in inhibiting adipocyte differentiation of the fat cells [18]. There are lots of benefits of other elements in DSW to health, which remain to be elucidated, particularly for the trace elements. The total amount of each element contained in DSW has been estimated [8], based upon the average concentration of each element in DSW. The total volume of DSW of 1.35 × 10^18^ m^3^ is shown in Table 1.

## 3. Potential Benefits of Deep Sea Water to Health

Many researchers and scientists have done studies about DSW, particularly about refined or balanced DSW. The minerals in it have been proven to improve many health problems. The potential health benefits of DSW are described below by providing some of the mechanisms involved. The findings that have been reviewed in this paper are significant, and comparisons have been made between the treated group and the control group.

### 3.1. Improvement of the Cholesterol Profiles

The most promising benefits that can be attained from DSW intake are that it is able to improve the cholesterol profiles in the serum and liver, respectively. Its applications have reduced the levels of triglyceride (TG), non-high-density lipoprotein cholesterol (non-HDL-C) levels, and total cholesterol (TC) in the serum and liver of animal models, respectively [4–6, 19–22, 24]. Drinking water produced from DSW which contains Mg of 600 and 1000 ppm, is able to decrease cholesterol levels by 18% and 15%, respectively [22]. Interestingly, a study of DSW consumption by hypercholesterolemic individuals proved that it could reduce TC and low density lipoproteins (LDL) and decreased lipid peroxidation in those subjects. The mechanisms for the improvement of cholesterol profiles are associated with the upregulation of hepatic low density lipoprotein receptor and cholesterol-7a-hydroxylase (CYP7A1) gene expressions, which are involved in cholesterol catabolism. A DSW intake resulted in a higher faecal cholesterol and bile acid excretions, thus decreasing the TC levels [5]. DSW decreases the lipid contents of hepatocytes through the activation of AMP-activated protein kinase, inhibiting the synthesis of cholesterol and fatty acid [19]. The details of respective studies are described in Table 3.

### 3.2. Protection from Cardiovascular Problems

DSW provides protection from cardiovascular diseases by decreasing the TC, TG, atherogenic index, and malondialdehyde (MDA) levels, while increasing the serum trolox equivalent antioxidant capacity (TEAC). The molecular mechanism of its cardiovascular protection is via upregulation of hepatic low density lipoprotein receptors (LDL receptors) and CYP7A1 gene expressions [5]. The cardioprotective effects of it were further proven, when its application can reduce abnormal cardiac architecture and apoptosis and enhance insulin-like growth factor-1 receptor (IGF-1R) cardiac survival signalling [25]. DSW can also improve cardiovascular hemodynamics in the study conducted by Katsuda et al. [2]. More details about the protective effects of DSW on the cardiovascular system are described in Table 4.

### 3.3. Prevention from Atherogenesis

Atherogenesis is the formation of plaque in the inner lining of an artery, which deposits fatty substances, cholesterol, cellular waste products, calcium, and other substances. Treatment with DSW was able to prevent the atherogenesis process [6, 21]. DSW with the hardness of 300, 900, and 1500 had significantly decreased the atherogenic index [(TC − HDL-C)/HDL-C] [5]. Antiatherogenic effects of DSW are associated with 5-adenosine monophosphate-activated protein kinase (AMPK) stimulation and the consequent inhibition of phosphorylation of acetyl-CoA carboxylase (ACC) [6]. AMPK plays an important role in lipid metabolism via the inhibition of 3-Hydroxy-3-methyl-glutaryl-CoA reductase (HMGCR) and ACC and then inhibits the production of cholesterol. The details of these studies are described in Table 5. Prevention of atherogenesis may avoid severe health problems, including coronary heart disease and stroke. DSW has antiatherogenic properties due to the existence of many beneficial mineral ions such as Mg and Ca in it. Hence, it could be widely promoted to enhance cardiovascular protection.

### 3.4. Reduction of Blood Pressure

DSW could improve cardiovascular hemodynamics and reduce blood pressure [2, 6, 20]. Hypertensive rats that were treated with DSW for eight weeks had lower blood pressure than the control group [20]. Reduced fats and blood lipids, such as in the artery, may be associated with the reduced blood pressure. Although DSW used in the study contains pretty much salt, the blood pressure did not increase. In another study [5], DSW application did not affect the blood pressure. Moreover, DSW can also prevent thrombotic disorder by suppressing the release of type-1 plasminogen activator inhibitor from the human vascular endothelial cells [7]. Lots of minerals combination in the DSW, such as Mg, Ca, and Na are associated with a reduced blood pressure. Na content may induce hypertension, though Mg supplement might lower the blood pressure by suppressing the adrenergic activity and, likely, natriuresis [46]. It is interesting that high Mg content can lower blood pressure in the presence of sodium. The details of these respective studies are described in Table 6.

### 3.5. Protection from Obesity

DSW has antiobesity properties and has been proven to reduce fat and body weight [1, 27, 29, 45]. It has been recognized as possible antiobesity therapeutics from nature [47]. The research has reported that DSW was significantly able to reduce lipids accumulation in the in vitro and in vivo models. Study with obese mice elucidated that DSW with hardness of 1000 was able to reduce body weight by 7%. It also increased the plasma protein levels of adiponectin and decreased plasma protein levels of resistin, RBP4, and fatty acid binding protein [1, 29]. The results suggest that the antiobesity activities were mediated by modulating the expression of obesity-specific molecules. The expression of key adipogenic genes such as peroxisome proliferator-activated receptor-*γ* (PPAR*γ*), CCAAT/enhancer-binding protein-*α* (C/EBP*α*), and adipocyte protein-2 (aP2) was suppressed, and the expression of glucose transporter 4 (GLUT4) was increased by its application [1, 27]. The magnificent effects of DSW on obesity were further proven when it stimulated mitochondrial biogenesis, the component which controls the release of energy associated with lipid metabolism [26]. Mg and Ca ions play a role as the main active components to reduce fats. However, DSW that has the same hardness of 1000 with drinking water which only contains Mg and Ca ions has showed small different effects in the obesity finding [13]. Thus, this hypothesized that Mg and Ca are not the main factors to reduce fats, as the roles of many elements in DSW remain to be elucidated. However the available findings on the clinical study showed that there is no significant difference of TG level and body weight, between treated subjects and controls [4]. More clinical studies are warranted. The detailed mechanisms involved regarding the effects of DSW on obesity-specific molecules are described in Table 7.

### 3.6. Treatment for Diabetes

DSW was able to improve glucose intolerance and suppress hyperglycaemia which indicated its ability to treat diabetes [26, 27, 29]. Its application had recovered the size of the pancreatic islets of Langerhans and increased the secretion of glucagon and insulin. Through quantitative reverse transcription polymerase chain reaction, DSW showed improvement results regarding the expression of hepatic genes involved in glycogenolysis and glucose oxidation. Whereas in muscles, glucose uptake, *β*-oxidation, and glucose oxidation were increased by its supplementation [29]. DSW increased the phosphorylation of IRS-1, LKB1, AMPK, and mTOR, which are signalling molecules related to lipid and glucose metabolism [27]. Moreover, blood glucose in treated mice was reduced by its application [27, 29]. The plasma glucose levels in DSW-fed mice were substantially reduced by 35.4%, compared to the control mice group [1]. The antidiabetic properties of it were associated with the existence of mineral ions such as Mg and Ca. The details of these studies are described in Table 8.

### 3.7. Treatment for Skin Problems

DSW is also capable of treating skin problems. In a study involving patients with atopic eczema/dermatitis syndrome (AEDS) treated with DSW, the improvement of skin symptoms such as inflammation, lichenification, and cracking of the skin was observed [31]. AEDS patients typically exhibit an imbalance of various essential minerals in hair, and some have toxic minerals present. From that study, DSW intake has restored the essential minerals such as Se and reduces the levels of toxic minerals such as mercury and lead in the treated patients. In another study, the intake of DSW has reduced allergic skin responses and serum levels of total IgE, Japanese cedar pollen-specific IgE, interleukin-4 (IL-4), IL-6, IL-13, and IL-18 in the patients with allergic rhinitis, compared to the distilled water intake which fails to give those effects [32]. In vivo study revealed that DSW can recover the atopic skin lesion by improving the skin symptoms such as edema, erythema, dryness, itching, transepidermal water loss (TEWL), decreased epidermal thickness, and infiltration of inflammatory cells. Its application can reduce allergic responses when reduction of total IgE levels and histamine released were recorded. It also inhibited upregulation of IgE, histamine, and proinflammatory cytokines (tumor necrosis factor *α* (TNF-*α*), IL-1*β*, and IL-6) in the serum. Downregulated CD4+/CD8+ ratio in spleen lymphocyte by 10% CDSW was also observed. Its application can reduce the expression of IL-4 and IL-10 from Th2 cells in the 10% CDSW-treated group [30]. The details of these studies are described in Table 9.

### 3.8. Protection from Hepatic Problems

High fat diets may cause problems to hepatic systems. DSW is able to give protection for hepatic problems. In a study by Chen et al. [33], it has decreased the lipid accumulation in livers, which are associated with the increase in daily faecal lipid and bile acid outputs. The hepatic antioxidative levels were also improved by its application, which were proven by the high capacity levels of liver glutathione and trolox equivalent antioxidant. DSW was able to regulate hepatic fatty acid homeostasis by upregulating genes related to b-oxidation of fatty acids, which are hepatic peroxisome proliferator-activated receptor-alpha, retinoid X receptor-alpha, and uncoupling protein-2 gene expression. Its application can attenuate hepatic damage, which is proven by reduced lipid peroxidation status in livers, which might be related to reducing hepatic malondialdehyde (MDA) content [33]. The liver damage indices which are aspartate aminotransferase (AST) and alanine aminotransferase (ALT) are also reduced by its application. The details of these studies are described in Table 10.

### 3.9. Treatment for Fatigue

DSW can restore fatigue and improve exercise workload. Its application has promoted the endurance and reduced exhaustive period of rats in an exercise test [34]. The ratios of lactic acid elimination to lactic acid increment were improved in DSW treated rats. The study showed low blood urea nitrogen (BUN) level of rats fed with D100 in a dosage of 30 mL/kg·d and D600 in dosages of 6, 12, and 30 mL/kg·d, respectively. As a result, the liver glycogen content had increased in the rats fed with D100 in a dosage of 6 mL/kg·d. Study regarding effects of DSW on human shows significant findings as well. DSW is able to accelerate recovery from physical fatigue of people, following an exhaustive physical challenge [35]. The findings suggested that DSW which has enriched contents of boron, magnesium, lithium, and rubidium may complement and enhance the molecular and cellular complexity of human during exercise, eradicate exercise-induced muscle damage, and strengthen antioxidant capability against oxidative stress. The details of these studies are described in Table 11.

### 3.10. Treatment for Stomach Ulcer

DSW can reduce ulcer area as well as apoptotic signalling in acetic acid-induced duodenal ulcers. It had upregulated antioxidant and enhanced Bcl-2 and thioredoxin reductase 1 expression in a study that used rats [36]. In that study, DSW ingestion provides intestinal protection via the antioxidant and antiapoptotic mechanisms of selenium. The details of this study are described in Table 12.

### 3.11. Prevention of Cancer

DSW is potential to prevent cancer. Its application can inhibit human breast cancer cell lines' migratory ability in a wound-healing assay. The inhibitory effects of DSW on breast cancer invasion/metastasis that uses MDA-MB-231 cells appears to be mediated through TGF-*β* and Wnt5a signalling, resulting in attenuated expression of CD44 [37]. In a study that uses the noninvasive MCF-7 cells, DSW treatment resulted in the inhibition of TPA-induced migration and MMP-9 activity with a concomitant decrease in mRNA levels of MMP-9, TGF-*β*, Wnt5a, and Wnt3a [37]. DSW also improves the quality of green tea prepared with it, in which it enhanced the production of epigallocatechin gallate (EGCG), which could potentially act as an inhibitor for N-nitrosation, which can induce mutagenic and cell damaging reactions [38]. The details of the studies regarding effects of DSW on cancer are described in Table 13.

### 3.12. Improvement in Antibacterial Activity

DSW has promising effects on antibacterial activity. The findings of its antibacterial activities were proven in the studies using the in vitro, in vivo, and clinical model as described in Table 14.

### 3.13. Treatment for Cataract

DSW application can delay cataract development [40, 41]. This effect is associated with the presence of Mg and Ca content in DSW. The details of these studies are described in Table 15.

### 3.14. Recovery from Osteoporosis

DSW has therapeutic potential on osteoporosis. DSW at hardness 1000 showed significant increase in proliferation of osteoblastic cell (MC3T3). In the in vivo study that uses DSW for 4 months, bone mineral density (BMD) was strongly enhanced followed by the significantly increased trabecular numbers through micro-CT examination. Biochemistry analysis showed that serum alkaline phosphatase (ALP) activity was decreased. BMSCs treated with DSW showed increase of osteogenic differentiation markers such as BMP2, RUNX2, OPN, and OCN and enhanced colony forming abilities, compared to the control group. The results demonstrated the regenerative potentials of DSW on osteogenesis, showing that it could potentially be applied in osteoporosis therapy as a complementary and alternative medicine (CAM). The details of these studies are described in Table 16.

## 4. Effects of Deep Sea Water in the Liver and Kidney Status

From the available studies, DSW hardness which ranges from 0 to 1500 had caused no damage to liver and kidneys. In a study, through in vivo and clinical subjects, ALT, AST, and BUN levels showed that there is no significant difference between treated subjects and the controls. The details of these respective studies are described in Table 17.

## 5. Functional Deep Sea Water with Other Substances

DSW is very beneficial to health. Its uses are applied to many DSW by-products. For example, it can enhance the antibacterial activity of yogurt [44]. The green tea leaves that were soaked in DSW had an increase in the antioxidant and catechin properties [38]. These findings increase the value of DSW as a health-promoting water. Combination of DSW with* Sesamum indicum* leaf extract (SIE) had prevented high fat diet-induced obesity, through AMPK activation in the visceral adipose tissue [45]. Furthermore, DSW has advantages for the development of functional fermentation food. The main factors of its increased health properties are due to it being able to increase functional metabolite production, intrinsic health functions of DSW, and the microbial use of mechanisms of converting the absorbed inorganic ions into highly bioavailable organic ions for the human body [48]. The detailed reviews regarding effects of DSW applications for the development of functional fermentation food are explained by Lee [48]. The detailed studies of functional deep sea water with other substances are described in Table 18.

## 6. Discussion and Conclusion

DSW originates from deep levels of the sea, which are far from contamination except for the natural occurrence of hazardous chemicals such as arsenic and mercury. It will usually undergo a process such as desalination to make it suitable for a particular purpose such as drinking water. The hardness of DSW of up to 1500 caused no cytotoxicity effects in the in vitro study [13]. However, the maximum hardness of it for human consumption should be remarked. The hardness values of water were estimated according to the following equation:

(1) $$\begin{array}{c} \text{Hardness}=Mg\left(\;\frac{mg}{L}\;\right)\times 4.1+Ca\left(\;\frac{mg}{L}\;\right)\times 2.5; \end{array}$$

see [49].

The probability of physical, chemical, or bacteriological contaminants present in the drinking water has triggered compulsory actions by most authorities to ensure that the water is subjected to appropriate treatments prior to being supplied. This includes the step of adding chlorine into the drinking water as a treatment. However, chlorine causes an unpleasant taste and raises health concerns such as cancer due to its ability to accumulate within the body [50–52]. Nowadays, it is becoming a trend to supply drinking water, through a vending machine that has the reverse osmosis system, from the treated wastewater and from the treated water pipeline. The process of water treatment will commonly cause reduction or loss of minerals. The increase in the availability of treated drinking water through processes such as reverse osmosis and chlorination should be put in high concern. Chlorine is not good for health. Furthermore, low nutrient in the drinking water can pose as a health threat to people that have nutrient deficiency. The desalinated DSW is usually added or concentrated with minerals, by the process of dilution, blending, or mixing it with concentrated minerals from the DSW [2, 4, 19, 53]. These mineralized methods of desalinated seawater have been a popular method. Therefore, the desalinated DSW will normally regain its minerals that might have been lost through the desalination process again, compared to the packaged drinking water, which has lost most of its minerals through water treatments. Thus, DSDW is able to have numerous minerals constituents in the water compared to the common mineral water sources such as aquifer, which only contain minerals that originated readily from the source. It can be claimed that the mineral contents in the DSW are greater than in the groundwater sources.

Through the impressive findings of DSW benefits to health, it is suggested that its utilization should be promoted widely. The nutrients deficiency of population in a region could be provided with DSW. Adequate nutrient contents in the drinking water supply can contribute to a healthy population status in the area of supply. Areas which have lack of nutrient contents in the water supply are linked with the deficiency of nutrients among their populations. Nutritious water supply is crucial for the people. Prevalence of cardiovascular mortality and sudden death is 10% to 30% greater in the soft water areas, which has low Mg or Ca ions, compared to the hard water areas that have high Mg or Ca ions in the water supply [54]. Intake of hard water has potential to decrease the risks of cardiovascular disease [55]. The importance of mineral contents in the drinking water is proven, when its intake is able to reduce calcium oxalate stone in the kidney of people that consume drinking water rich in minerals such as Mg, Ca, and bicarbonate [56–58]. In contrast, consumption of low calcium content in the drinking water has resulted in the hip fracture incident in the Norwegian population [59]. Instead of epidemiological studies, researchers have identified the importance of mineral water content in the experimental studies. According to the study, the rabbits and men which consumed water with low mineral contents have higher risks of cardiovascular disease, compared to the group that consumed water with high mineral content [60]. The miracle of water to cure diseases has progressively been discussed. One of the mechanisms of mineral water to treat diseases is through the existence of minerals which are capable of activating the aquaporin genes, which are responsible for transporting water within the cells [61]. Lack of aquaporin gene activation has been linked to many disease occurrences [62]. Minerals in the DSW are plenty and thus could be a major factor in curing diseases.

Some areas may have lack of nutrients in the soil and crops, which may pose as health threats to its consumers. The soil provides minerals to the plants, and through the plants the minerals go to the animals and humans [63]. Referring to the chain, it is a health threat to people that usually rely on the crops and animals as their main nutrients provider. For instance, nutrient deficiency in the land of South Africa was associated with many diseases occurrences such as thyroid, iodine deficiency disorders (IDD), Mseleni Joint Disease (MJD), HIV-AIDS, and Mg insufficiency [64]. Besides that, the groundwater could be contaminated with man-made activities including the industries, agriculture, and logging. These could pose as a threat to the residents that use groundwater as a source for drinking water. For instance, agricultural activities have caused an increase in the nitrate concentration of groundwater in the area of Machang, Malaysia, resulting from the fertilizer application [65]. DSW which is far from man-made contamination could provide a safe water source. DSW is rarely polluted, has no or slight bacteria existence, and is very pure [2, 8, 66].

Furthermore, nutrients deficiency among the people was also associated with the types of daily food intake. For instance, the regular consumption of phytate content foods had caused the zinc deficiency among Korean people [67]. Phytate impairs the zinc bioavailability. Thus, choosing the right foods is crucial for nutrient intake. Dynamic activities in today's life had caused the tendency for people to choose fast, instant, and easy prepared foods. These kinds of foods usually contain a small amount of nutrients, which is not the most promising source of nutrients intake. Minerals in food may also be lost during cooking [68–70]. In a nutshell, nutrients intake should not solely rely on food intake. DSW has lots of minerals to supply, and could be provided in the form of health drinks or water supply, as an alternative to maintain nutrients source. The roles of minerals in the water to heal disease and maintain health has already been recognized. Water can be classified into a few categories based on its total salt content, its mineral biological activity, and its ion mineral composition [71]. The effort to put DSW as a water source that is beneficial for health should be enhanced. The studies regarding types of mineral water have also been progressively carried out. Examples of these studies can be referred to Astel et al. [72], included in the discussion about the types of minerals available in the water, types of available water treatments, associated regulations, and therapeutic potentials of mineral water. The study to classify DSW into particular types of water based on the types of production should be established, as there is a great therapeutic potential about it yet to be discovered.

Ideally, countries with the accessibility to pump up water from DSW should consider maximizing the use of it. Perhaps, the only limitations are the technology provider and cost of production, rather than reachable sources to the DSW itself. Technologies that are involved may include desalination, low vacuum temperature, and ocean thermal energy conversion (OTEC). OTEC is a kind of technology which could produce water as a by-product from its process, without the extensive cost [73]. There are many great findings from the studies regarding DSW applications in the in vitro models such as using 3T3L-1 cells, and in the in vivo models such as using mice, and rabbits. However, the potential health benefits of its applications in the clinical studies are not widely established. Hence, the study of its applications especially to the human health should be conducted more. DSW is worthy of further investigations and could be developed as medicated water in the prevention and treatment of many health problems, especially lifestyle-related diseases.

## Figures and Tables

**Table 1:** Total amount of elements in deep sea water [8].

Element	Total (10^6^ ton)
CI	26,120,000,000
Na	14,550,000,000
Mg	1,728,000,000
S	1,312,000,000
Ca	556,000,000
K	538,000,000
Br	90,000,000
C	36,000,000
N	11,700,000
Sr	10,500,000
B	6,100,000
O	3,800,000
Si	3,800,000
F	1,900,000
Ar	840,000
Li	240,000
Rb	160,000
P	84,000
I	78,000
Ba	20,000
Mo	14,000
U	4,300
V	2,700
As	1,600
Ni	650
Zn	470
Kr	420
Cs	413
Cr	271
Sb	270
Ne	216
Se	209
Cu	202
Cd	94
Xe	89
Fe	40
Al	40
Mg	27
Y	22
Zr	20
TI	17
W	13
Re	11
He	10
Ti	8.8
La	7.6
Ge	2.4
Nb	<7
Hf	4.6
Nd	4.4
Ta	<3
Ag	2.7
Co	1.6
Ga	1.6
Er	1.6
Yb	1.6
Dy	1.5
Gd	1.2
Pr	0.9
Ce	0.9
Se	0.9
Sm	0.8
Sn	0.7
Ho	0.5
Lu	0.3
Be	0.3
Tm	0.3
Eu	0.2
Hg	0.2
Rh	0.1
Te	0.1
Pd	0.008
Pt	0.07
Bi	0.04
Au	0.03
Th	0.02
In	0.01
Ru	<0.006
Os	0.003
Ir	0.0002

**Table 2:** Amount of elements in the surface seawater and deep sea water [6].

Type of element	Surface seawater (mg/L)	Deep sea water (mg/L)
Na	10800	7240
K	392	10400
Ca	411	39
Mg	1290	96100
Sr	8.1	0.17
B	4.45	320
Fe	0.003	0.25
Li	0.17	11.7
Cu	0.0009	0.22
Co	0.0004	0.26
Mo	0.01	0.62
Ni	0.0066	0.11
Cr	0.0002	0.087
Rb	0.12	1.2
Si	2.9	0.5
V	0.002	1.2
F	13	21.8
Br	67.3	5400
I	0.064	5.5

**Table 3:** Effects of deep sea water on cholesterol levels.

Type of study model	Experimental method [subject (age/weight), treatment dosage, duration of treatment]	Major activity	Mechanism of action	Reference
In vivo study	High fat diet (HFD) male Wistar rats (200–220 g), DSW 1,000 hardness, ad libitum, 4 weeks	Increased the level of HDL-C.	ND.	[19]

In vivo study	Cholesterol-fed diet (CFD) male New Zealand white rabbits (1500–2000 g) fed diet containing 3.75, 37.5, and 75 mg/kg of Mg, DSW 1410 hardness, 8 weeks	Improved plasma total cholesterol (TC), triglyceride (TG), and LDL-C levels.	Improved the protein expression of AMPK phosphorylation, ACC phosphorylation, and HMGCR.	[6]

In vivo study	High cholesterol diet (HCD) ICR mice (7 weeks), reverse osmosis (RO DSW) (44.6 hardness), electrodialysis (ED DSW) (4685.9 hardness) and 10% (v/v) dilution with ddH_2_O 10% DSW (544.2 hardness), ad libitum, 8 weeks	Reduced the level of TG, TC, and non-high-density lipoprotein cholesterol (non-HDL-C) levels in the serum and liver of animal models, respectively.	Increase in daily faecal lipid of TG and TC and bile acid outputs.	[20]
	HFD Hamster (5 weeks), DSW 300, 900, 1500 hardness, ad libitum, 6 weeks		Increase in daily faecal lipid of TG and TC and bile acid outputs. Upregulated hepatic low-density-lipoprotein receptor (LDL receptor) and cholesterol-7a-hydroxylase (CYP7A1) gene expressions.	[5]

Type of study model	Male hyperlipidemia rabbits (1.8–2.0 g), DSW 1200 hardness, 150 ml/d, ad libitum, 12 weeks	Reduced plasma TC and plasma LDL cholesterol level. Increased plasma HDL cholesterol.	ND.	[21]

In vivo study	Male Wistar rats (90 g), DSW containing 200, 600, and 1000 mg/L of Mg, ad libitum, 4 weeks	Attenuated plasma TC.	ND.	[22, 23]

Clinical study	Hypercholesterolemic individuals (23 men and 19 women), DSW (1410 hardness), supplemented 1050 mL daily, 6 weeks	Decreased serum TC and low-density lipoprotein cholesterol (LDL-C).	Decreased lipid peroxidation in hypercholesterolemic subjects.	[4]

Clinical study	CFD and hyperlipemia male Japanese rabbits, DSW hardness of 28, 300, and 1200, 150 ml/d, ad libitum, 4 weeks	Reduced TC and LDL-C levels in hyperlipemia rabbits. Prevented increase of TC and LDL-C levels in CFD rabbits.	ND.	[24]

ND: not described.

**Table 4:** Effects of deep sea water on cardiovascular protection.

Type of study model	Experimental method [subject (age/weight), treatment dosage, duration of treatment]	Major activity	Mechanism of action	Reference
In vivo study	HCD ICR mice (7 weeks), reverse osmosis-DSW 44.6 hardness, Electrodialysis-DSW 4685.9 hardness, 10% DSW 544.2 hardness, 8 weeks	Reduced abnormal cardiac architecture, apoptosis in left ventricle (LV). Increased cardiac survival signalling components in LV of mice. Change in Fas and mitochondrial-dependent apoptotic components in LV of mice. Change in apoptosis related proteins and cardiac apoptotic cells in LV of mice.	Decreased LV diameter, LV thickness, and ratio of thickness to diameter in hearts. Increased insulin-like growth factor-1 receptor, phosphoinositide-3-kinases, and p-AKT/AKT ratio. Decreased the protein products of TNF-*α* in LV of mice. Decreased levels of Fas, cytochrome c, cleaved caspase-9, t-Bid, and cleaved caspase-3. Decreased Bak and increased antiapoptotic proteins, including Bcl-XL and ratio of p-Bad to Bad. Decreased TUNEL-positive cardiac cells.	[25]

In vivo study	High fat/cholesterol-fed (HFCD) male Syrian Golden hamster (5 weeks), DSW 300, 900, and 1500 hardness, ad libitum, 6 weeks	Decreased levels of serum TC, TG, atherogenic index, and malondialdehyde.	Increase in daily faecal lipid of TG and TC and bile acid outputs. Upregulated hepatic low-density-lipoprotein receptor (LDL receptor) and cholesterol-7a-hydroxylase (CYP7A1) gene expressions. Increase of serum trolox equivalent antioxidant capacity (TEAC).	[5]

In vivo study	Kurosawa and Kusanagi-Hypercholesterolemic (KHC) rabbits (4 months), DSW 1000 hardness, 500 ml/d, 6 months	Improved cardiovascular hemodynamics.	Lowered systolic, diastolic pulse, mean arterial pressures, and total peripheral resistance.	[2]

**Table 5:** Effects of deep sea water on atherosclerosis.

Type of study model	Experimental method [subject (age/weight), treatment dosage, duration of treatment]	Major activity	Mechanism of action	Reference
In vivo study	CFD male New Zealand white rabbits (1500–2000 g) fed diet contain 3.75, 37.5, and 75 mg/kg of Mg, DSW 1410 hardness, 8 weeks	Reduced serum lipids, prevented atherogenesis, and suppressed serum cholesterol levels. Reduced lipids accumulation in liver tissues, and limited aortic fatty streaks.	Improved protein expression of AMPK phosphorylation, ACC phosphorylation, and HMGCR.	[6]

In vivo study	Male hyperlipidemia rabbits (1.8–2.0 g), DSW 1200 hardness, 150 ml/d, ad libitum, 12 weeks	Suppressed lipid deposition on the inner wall of the aorta. Suppressed foam cell formation.	Reduced plasma TC, plasma LDL cholesterol, and LPO. Increased plasma HDL cholesterol. Increased glutathione peroxidase (GPx) activity. Decreased plasma lipid peroxide (TBARS) value.	[21]

In vivo study	CFD and hyperlipemia male Japanese rabbits, DSW hardness of 28, 300, and 1200, 150 ml/d, 4 weeks	Reduced TC and LDL-C levels in hyperlipemia rabbits. Prevented increase of TC and LDL-C levels in CFD rabbits. Reduced lipid accumulation in liver and permeation of macrophages in CFD rabbits.	ND.	[24]

ND: not described.

**Table 6:** Effects of deep sea water on blood pressure.

Type of study model	Experimental method [subject (age/weight), treatment dosage, duration of treatment]	Major activity	Mechanism of action	Reference
In vivo study	Spontaneous hypertensive rats (250–300 g) fed diet containing 3.75, 37.5, and 75 mg/kg of Mg, DSW 1410 hardness, ad libitum, 8 weeks	Decreased blood pressure.	Decreased systolic and diastolic pressure.	[6]

In vivo study	Kurosawa and Kusanagi-Hypercholesterolemic (KHC) rabbits (4 months), DSW 1000 hardness, 500 ml/d, 6 months	Decreased blood pressure.	Lowered systolic, diastolic pulse, and mean arterial pressure and total peripheral resistance.	[2]

**Table 7:** Effects of deep sea water on obesity.

Type of study model	Experimental method [subject (age/weight), treatment dosage, duration of treatment]	Major activity	Mechanism of action	Reference
In vitro study	C2C12 cells, DSW 100, 500, 1000, 1500, and 2000 hardness, indicated time of 0, 1, 2, and 3 days	Increased mitochondrial biogenesis and function.	Enhanced gene expression of peroxisome proliferator-activated receptor gamma coactivator 1 *α* (PGC-1a), nuclear respiratory factor-1 (NRF-1), and mitochondrial transcription factor A (TFAM); mitofusin-1/2 (MFN1/2) and dynamin-related protein 1 (DRP1) for mitochondrial fusion; optic atrophy 1 (OPA1) for mitochondrial fission; translocase of outer mitochondrial membrane 40 (TOMM40) and translocase of inner mitochondrial membrane 44 (TIMM44) for mitochondrial protein import; carnitine palmitoyltransferase 1*α* (CPT1*α*) and medium-chain acyl-CoA dehydrogenase (MCAD) for fatty acid oxidation; and cytochrome c (CytC) for oxidative phosphorylation. Increased mitochondria staining, citrate synthase (CS) activity, CytC oxidase activity, NAD+ to NADH ratio, and the phosphorylation of signalling molecules such as AMPK and sirtuin 1 (SIRT1).	[26]

In vitro study	3T3-L1 cells, DSW 100, 500, and 1000 hardness, 3 days	Decreased lipid accumulation.	Reduced expression mRNA levels of PPAR*γ* and C/EBP*α* and protein levels of fatty acid binding protein and adiponectin.	[13]

In vivo study	HFD C57BL/6J mice (6 weeks), DSW 500, 1000, and 2000 hardness, ad libitum, 20 weeks	Enhanced mitochondrial biogenesis in muscles.	Improved mitochondrial DNA (mtDNA) content in the muscles of HFD-induced obese mice. Enhanced expression of PGC-1*α*, NRF1, and mtTFA. Enhanced estrogen-related receptor *α* (ERR*α*), PPAR*α*, and PPAR*δ*.	[26]

In vivo study	HFD C57BL/6J mice (6–26 weeks), DSW 500, 1000, and 2000 hardness, ad libitum, 20 weeks	Suppressed body weight gain. Inhibited increase in adipocyte size. Suppressed the expression of adipogenic, lipogenic, lipolytic, and proinflammatory cytokine genes. Increased the expression of adipokines and b-oxidation genes in fat.	Suppressed mRNA expression of key adipogenic genes such as PPAR*γ*, C/EBP*α*, and aP2. Increased the expression of GLUT4, adiponectin, and leptin. Decreased the expressions of IL-6 and TNF-a. Decreased the expressions of sterol regulatory element-binding protein 1c (SREBP1c) and fatty acid synthase (Fas), which are involved in lipogenesis; adipose triglyceride lipase (ATGL) and hormone-sensitive lipase (HSL), which are involved in lipolysis. Increased the expression of MCAD and CPT1*α*, which are involved in b-oxidation. Increased phosphorylation of IRS-1, LKB1, AMPK, and mTOR in fat.	[27]

In vivo study	Male C57BL/6J ob/ob mice, DSW 1000 hardness, ad libitum, 84 days	Decreased body weight gain by 7%. Reduced plasma glucose levels by 35.4%.	Increased glucose disposal. Increased plasma protein levels of adiponectin. Decreased plasma protein levels of resistin, RBP4, and fatty acid binding protein. Increased GLUT4 and AMP-activated protein kinase levels in skeletal muscle tissue. Decreased PPAR*γ* and adiponectin in adipose tissue.	[1]

**Table 8:** Effects of deep sea water on diabetes.

Type of study model	Experimental method [subject (age/weight), treatment dosage, duration of treatment]	Major activity	Mechanism of action	Reference
In vitro study	Differentiated C2C12 cells, DSW 100, 500, 1000, 1500, and 2000 hardness, 1 hr	Increased glucose uptake.	Stimulated the phosphorylation of IRS-1, LKB1, AMPK, and mTOR and improved impaired phosphorylation of these molecules.	[28]

In vitro study	Matured 3T3-L1 cells, DSW 500, 1000, and 2000 hardness, 1 hr	Increased glucose uptake.	Increased AMPK phosphorylation in 3T3-L1 pre- and mature adipocytes. Stimulated phosphoinositol-3-kinase and AMPK pathway-mediated glucose uptake.	[29]

In vivo study	Streptozotocin- (STZ-) induced diabetic male ICR mice (4–9 weeks), DSW 1000, 2000, and 4000 hardness, ad libitum, 4 weeks	Improved impaired glucose tolerance. Regulated blood glucose levels by inhibited glucose production and enhanced glucose uptake via regulation of gene expression.	Increased adiponectin and leptin levels and reduced the levels of the proinflammatory cytokines IL-6 and TNF-*α*. Improved architecture of pancreatic islets of Langerhans and enhanced insulin secretion from *β*-cells. Stimulated the phosphorylation of IRS-1, LKB1, AMPK, and mTOR and improved impaired phosphorylation of these molecules in muscle. Downregulated the expression of phosphoenolpyruvate carboxykinase (PEPCK) and glucose 6-phosphatase (G6Pase), both of which are required for gluconeogenesis; glucokinase (GK) and citrate synthase (CS), both of which are required for glucose oxidation; and liver glycogen phosphorylase (LGP), which is required for glycogenolysis. Upregulated glycogen synthase (GS) expression. Upregulated the expression of GLUT1 and GLUT4 in skeletal muscle, which are required for glucose transport; glucokinase and citrate synthase, which are required for glucose oxidation; and acyl-CoA oxidase (ACO), CPT1*α*, and MCAD, which are required for *β*-oxidation.	[28]

In vivo study	HFD-induced diabetic male C57BL/6J mice (6–25 weeks), DSW 500, 1000, and 2000 hardness, ad libitum, 20 weeks	Improved impaired glucose tolerance. Suppressed the expression of hepatic genes involved in glucogenesis, glycogenolysis, and glucose oxidation. Increased glucose uptake, *β*-oxidation, and glucose oxidation in muscle. Improved impaired AMPK phosphorylation in the muscles and livers.	Recovered size of the pancreatic islets of Langerhans and increased the secretion of insulin and glucagon. Increased adiponectin levels. Decreased IL-6 and TNF-*α* levels. Downregulated the expression of PEPCK and G6Pase for gluconeogenesis; GK and CS for glucose oxidation; and LGP for glycogenolysis. Upregulated the expression of GS for glycogenesis. Upregulated the GLUT1 and GLUT4 for glucose transport, GK and CS for glucose ACO, CPT1*α*, and MCAD for *β*-oxidation in skeletal muscle. Increased the expression of SIRT family proteins such as SIRT1, SIRT4, and SIRT6.	[29]

In vivo study	Male C57BL/6J ob/ob mice, DSW 1000 hardness, ad libitum, 84 days	Reduced glucose levels in plasma by 35.4%.	Increased glucose disposal. Increased adiponectin levels in plasma. Decreased plasma protein levels of resistin, RBP4, and fatty acid binding protein. Increased GLUT4 and AMP-activated protein kinase levels in skeletal muscle tissue.	[1]

**Table 9:** Effects of deep sea water on skin diseases.

Type of study model	Experimental method [subject (age/weight), treatment dosage, duration of treatment]	Major activity	Mechanism of action	Reference
In vivo study	Male NC/Nga mice (6 weeks), 2% concentrated DSW (CDSW) (7958.6 hardness), 10% CDSW (39793 hardness), 200 *μ*L of test samples, five times per week, six weeks	Reduced severity of symptoms in the skin lesions, such as edema, erythema, dryness, itching, and transepidermal water loss (TEWL). Decreased epidermal thickness and infiltration of inflammatory cells.	Inhibited upregulation of IgE, histamine, and proinflammatory cytokines (TNF-*α*, IL-1*β*, and IL-6) in the serum. Downregulated CD4+/CD8+ ratio in spleen lymphocyte by 10% CDSW. Reduced the expression of IL-4 and IL-10 from Th2 cells in the 10% CDSW-treated group.	[30]

Clinical study	33 patients (mean age 26 years, range 1–50 years, 13 male and 20 female subjects), DSW 1000 hardness, 500 ml/day, 6 months	Improved skin symptoms. Balanced certain minerals in the body.	Improved skin symptoms such as inflammation, lichenification, and cracking in skin. Restored essential minerals such as Se and reduced the level of toxic minerals such as mercury and lead.	[31]

Clinical study	50 patients with allergic rhinitis (age 22–50 years), DSW 1000 hardness, 500 ml/day, 3 weeks	Improved skin symptoms.	Reduced allergic skin responses and serum levels of total IgE, Japanese cedar pollen-specific IgE, IL-4, IL-6, IL-13, and IL-18.	[32]

**Table 10:** Effects of Deep Sea Water on Hepatic Protection.

Type of study model	Experimental method [subject (age/weight), treatment dosage, duration of treatment]	Major activity	Mechanism of action	Reference
In vitro study	HepG2 cells, DSW 200, 400, 600, 800, and 1000 hardness, 24 hr	Decreased lipids accumulation.	Inhibited the activity of HMGCR by 30.2%. Increased the phosphorylation level of AMPK by 15.2%. Reduced p68 of SREBP-1 levels by 55%. DSW of hardness 600, 800, and 1,000 increased p68 levels of SREBP-2 by 12, 42, and 80%, respectively. DSW of hardness 600, 800, and 1,000 increased level of CYP7A1 by 41, 115, and 162%, respectively. DSW of hardness 1,000 increased Apo AI content by 20.3%.	[19]

In vivo study	HFD male Wistar rats (200–220 g), DSW 1,000 hardness, ad libitum, 4 weeks	Decreased levels of TC and TG in liver. Improved liver function.	Decreased serum levels of AST and ALT.	[19]

In vivo study	HFD C57BL/6J mice (6–26 weeks), DSW 500, 1000, and 2000 hardness, ad libitum, 20 weeks	Suppressed the expression of genes involved in lipogenesis and cholesterol synthesis; and increased the expression of genes related to b-oxidation in liver. Improved severe liver steatosis. Regulated mitochondrial biogenesis and function in liver.	Decreased the expression of Fas and acetyl-CoA carboxylase 1 (ACC1), which are involved in lipogenesis, and liver X receptor a (LXR a), and 5-hydroxy-3-methylglutaryl-coenzyme A reductase (HMG-CoAR), which are involved in cholesterol metabolism. Increased the expression of MCAD and CPT1*α*, which are involved in b-oxidation. Increased the phosphorylation of IRS-1, LKB1, AMPK, and mTOR in liver. Increased expression of PGC1a, NRF1, Tfam, and mtDNA content in liver.	[27]

In vivo study	HFD male Golden Syrian hamsters (5 weeks), DSW 300, 900, and 1500 hardness, ad libitum, 6 weeks	Decreased lipids accumulation in liver. Regulated hepatic fatty acid homeostasis. Improved hepatic antioxidative levels. Attenuated hepatic damage.	Increased daily faecal lipid and bile acid outputs. Upregulated genes of hepatic PPAR*α*, retinoid X receptor-alpha, and uncoupling protein-2 (UCP-2) gene expression. Maintained higher liver glutathione and TEAC levels. Reduced lipid peroxidation status (MDA content) in liver.	[33]

**Table 11:** Effects of deep sea water on fatigue.

Type of study model	Experimental method [subject (age/weight), treatment dosage, duration of treatment]	Major activity	Mechanism of action	Reference
In vivo study	Exercise-induced fatigue male Wistar rats, DSW 100, and 600 hardness, dosages (6, 12, and 30 mL/kg·d)	Promoted the endurance of rats in exercise test. Reduced exhaustive period.	Improved the ratio of lactic acid elimination to lactic acid increment. Reduced BUN level of rats fed with D100 in a dosage of 30 mL/kg·d and D600 in dosages of 6, 12, and 30 mL/kg·d. Increased liver glycogen content in rats group fed with D100 in a dosage of 6 mL/kg·d.	[34]

Clinical study	12 healthy male volunteers (age 24 ± 0.8 years; height 171.8 ± 1.5 cm; weight 68.2 ± 2.3 kg; VO_2_max 49.7 ± 2.2 ml·kg^−1^·min^−1^), randomized, double-blind, placebo-controlled, DSW 710 hardness, fatiguing exercise conducted for 4 hr at 30°C	Accelerated recovery from physical fatigue.	Complete recovery of aerobic power within 4 hr. Elevated muscle power above placebo levels within 24 hr. Increased circulating creatine kinase (CK) and myoglobin; indicatives of exercise-induced muscle damage, were completely eliminated, in parallel with attenuated oxidative damage.	[35]

**Table 12:** Effects of deep sea water on stomach ulcer.

Type of study model	Experimental method [subject (age/weight), treatment dosage, duration of treatment]	Major activity	Mechanism of action	Reference
In vivo study	Female Wistar rats (220–250 g weight), DSW 600 (41 mL/day), DSW 1200 (39 mL/day), 1 week	Reduced ulcer area as well as apoptotic signalling in acetic acid-induced duodenal ulcers. DSW influenced oxidative stress genes expression. Upregulated antioxidant and antiapoptotic genes and downregulated proapoptotic gene expression by DSW of hardness 600 and 1200, respectively.	Increased pH value, scavenging H_2_O_2_, and HOCl activity and reduced ORP value. Enhanced Bcl-2 and thioredoxin reductase 1 expression. DSW1200 activated the expression of flavin-containing monooxygenase 2 (Fmo2), Gpx1, Gpx5, Gpx6, glutathione reductase (Gsr), nitric oxide synthase 2, inducible (Nos2), thioredoxin reductase 1 (Txnrd1), superoxide dismutase 1 (Sod1), some antioxidant-related genes, peroxiredoxin 4 (Prdx4), and selenoprotein P plasma 1 (Sepp1). DSW600 and DSW1200 upregulated Txnrd1 and Bcl-2 and downregulated Bax, caspase 3, and PARP in duodenal cells. DSW 600 upregulated expression of apoptosis-inducing factor, mitochondrion-associated 1 (Aifm1), DNA-damage-inducible, alpha (Gadd45a), myeloid cell leukemia sequence 1 (Mcl 1), and X-linked inhibitor of apoptosis (Xiap). DSW 600 downregulated expression of apoptosis inhibitor 5 BCL2-associated athanogene (Api5), cell death-inducing DFFA-like effector b (Ciedb), cytochrome c, and somatic (Cycs), Fas (TNF receptor superfamily, member 6), growth arrest and mitogen activated protein kinase 1 (Mapk1), PYD and CARD domain containing (Pycard). DSW 1200 upregulated expression of Fas, Gadd45a, and Mcl1. DSW 1200 downregulated expression of Aifm1, Api5, Bag1, Cideb, Cycs, and Pycard.	[36]

**Table 13:** Effects of deep sea water on cancer.

Type of study model	Experimental method [subject (age/weight), treatment dosage, duration of treatment]	Major activity	Reference
In vitro study	MDA-MB-231 cells, DSW 200, 400, 800, and 1500 hardness, 2-3 days	Inhibited cells' migratory ability in a wound-healing assay, mediated through TGF-*β* and Wnt5a signalling, resulting in attenuated expression of CD44.	[37]

In vitro study	Noninvasive MCF-7 cells, DSW 200, 400, 800, and 1500 hardness, 2-3 days	Inhibited TPA-induced migration and MMP-9 activity with a concomitant decrease in mRNA levels of MMP-9, TGF-*β*, Wnt5a, and Wnt3a.	[37]

	Green tea leaves were soaked in desalinated DSW at 75°C for 10 min	Increased nitrite scavenging activity from 31.33 ± 0.05 to 37.12 ± 0.42%. Increased overall amounts of catechins.	[38]

	*Salmonella Typhimurium* TA98 and TA100, Ames test, methanol extract of kochujang added with sea tangle and deep sea water salts (SDK), 200 *μ*g/plate	71.4% inhibitory effect on the mutagenesis induced by 4NQO against TA98 strain. 56.1% and 83.6% inhibitions on the mutagenesis induced by 4NQO and MNNG against TA100 strain.	[39]

**Table 14:** Effects of deep sea water on cataract.

Type of study model	Experimental method [subject (age/weight), treatment dosage, duration of treatment]	Major activity	Mechanism of action	Reference
In vivo study	Male Shumiya cataract rat (5–15 weeks), DSW (Mg^2+^, 200 mg/L, Ca^2+^; 71 mg/L), 9-10 weeks	Delayed cataract development.	Reduced less opaque and nitric oxide (NO) levels.	[40]

In vivo study	Male Shumiya cataract rat (5–15 weeks), DSW containing Mg of 50, 200, and 1000 mg/L, respectively, 9-10 weeks	Delayed cataract onset.	Mg suppressed Ca influx into the lens.	[41]

**Table 15:** Effects of deep sea water on antibacterial activity.

Type of study model	Experimental method [subject (age/weight), treatment dosage, duration of treatment]	Major activity	Reference
In vitro study	Five types ratio of DSW containing magnesium : calcium (Mg : Ca) ratios of 1 : 2 (A), 1 : 1 (B), 3 : 1 (C), 1 : 0 (D), and 0 : 1 (E) at different concentrations to give levels of hardness of 100, 250, 500, and 1000; produced 20 types of samples Sixteen *H. pylori* strains, clinical isolates were obtained from patients with gastric cancer, gastric ulcer, and normal gastric mucosa	Inhibited bacterial growth and mobility.	[42]

In vitro study	Sheep blood, *H. pylori* obtained from gastric biopsy specimens of peptic ulcer patients, 3 to 5 days	DSW hardness of 1200 and 2400 inhibited growth of *H*. *pylori* strains by 20% and 60%, respectively.	[36]

In vivo study	Male Mongolian gerbils (4 weeks), DSW at 5 different Mg/Ca ratios (hardness of 1000) were administered for 2 weeks	Decreased amount of *H. pylori* colonized in stomach by treatment with 2 types of DSW ratio which are C and D. Anti-*H. pylori* effects were observed in ≥90% of subjects.	[42]

Clinical study	Healthy subjects infected with *H. pylori*, DSW at 5 different Mg/Ca ratios (hardness: 1000), 1 L/daily, 10 days	Reduced Δ13 C values.	[42]

**Table 16:** Effects of deep sea water on osteoporosis.

Type of study model	Experimental method [subject (age/weight), treatment dosage, duration of treatment]	Major activity	Reference
In vitro study	Osteoblastic cell (MC3T3), DSW 50, 1000, and 2000 hardness, 3 days	Increased cells proliferation.	[43]

In vitro study	Bone marrow stromal cells (BMSCs), DSW 1000 hardness, 3 days	Enhanced colony forming abilities.	[43]

In vivo study	Ovariectomized (OVX) SAMP8 mice (4 months), DSW 1000 hardness, 5.2 mL/day, 4 months	Enhanced bone mineral density. Increased trabecular numbers through micro-CT examination. Decreased serum alkaline phosphatase (ALP). Increased osteogenic differentiation markers such as BMP2, RUNX2, OPN, and OCN.	[43]

**Table 17:** Effects of deep sea water in the liver and kidney status.

Type of study model	Experimental method [subject (age/weight), treatment dosage, duration of treatment]	Major activity	Reference
In vivo study	HFD male Wistar rats (200–220 g), DSW 1,000 hardness, ad libitum, 4 weeks	Improved liver function by the decrease of serum levels of AST and ALT.	[19]

In vivo study	HFD male Golden Syrian hamsters (5 weeks), DSW 300, 900, and 1500 hardness, ad libitum, 6 weeks	Attenuated serum AST values in hamsters drinking DSW 300, 900, and 1500. Lower serum ALT values in hamsters drinking DSW 900 and DSW 1500.	[33]

In vivo study	CFD male New Zealand white rabbits (1500–2000 g) fed diet containing 3.75, 37.5, and 75 mg/kg of Mg, DSW 1410 hardness, 8 weeks	No differences were observed in values of AST and ALT.	[6]

In vivo study	Male hyperlipidemia rabbits (1.8–2.0 g), DSW 1200 hardness, 150 ml/d, ad libitum, 12 weeks	No differences were observed in values of AST and ALT.	[21]

Clinical study	Hypercholesterolemic individuals (23 men and 19 women), DSW (1410 hardness), supplemented 1050 mL daily, 6 weeks	No significant difference of ALT, AST, and BUN levels between treated subjects and controls.	[4]

**Table 18:** Effects of functional deep sea water with other substances.

Type of study model	Experimental method [subject (age/weight), treatment dosage, duration of treatment]	Major activity	Mechanism of action	Reference
In vivo study	Outbred albino female ICR mice (20–26 g), yogurt containing DSW, 10.3 g hardness of CaCO_3_/L, 8 weeks	Increased populations of intestinal lactic acid bacteria. Decreased the activity of serum AST and ALT. Reduced TC, TC to HDL-C ratio, TAG, and HDL-C in serum.	ND.	[44]

In vivo study	HFD-induced obesity ICR (4 weeks), DSW, and DSW + 125 mg/kg SIE (DSS), ad libitum, treated with SIE once per day for 8 weeks	Reduced body weights in the DSW group by 3.95% and in the DSS group by 8.42%, respectively. Decreased plasma glucose levels in the DSW group by 14.9% and in the DSS group by 36.4%, respectively. Decreased serum levels of glucose, TAG, and leptin. Decreased insulin resistance index (HOMA-IR) values for the DSS-treated group by 38.2%.	Decreased size of the epididymal white, retroperitoneal white, and scapular brown adipose tissue. Increased levels of phosphorylated AMPK and its substrate and ACC in mice epididymal adipose tissues. Upregulated the expression levels of lipolysis-associated mRNA, PPAR-*α*, cluster of differentiation 36 (CD36), and energy expenditure-associated mRNA and UCP2 and CPT1 epididymal adipose tissues. Suppressed the expression of SREBP1 at the mRNA level.	[45]

In vivo study	Green tea leaves were soaked in desalinated DSW at 75°C for 10 min	Increased antioxidant activity.	Increased 2, 2-diphenyl-1-picrylhydrazyl (DPPH) radical scavenging activities by 83.98% and increased reducing power by 15%. Increased nitrite scavenging activity from 31.33 ± 0.05 to 37.12 ± 0.42%. Increased amounts of catechins and caffeine.	[38]

ND: not described.
